# A prognostic model constructed by ferroptosis-associated genes (FAGs) in papillary renal cell carcinoma (PRCC) and its association with tumor mutation burden (TMB) and immune infiltration

**DOI:** 10.1007/s12094-024-03617-y

**Published:** 2024-08-16

**Authors:** Yong-Bo Chen, Xin Yang, Dong Lv, Liang-You Tang, Ying-Wen Liu

**Affiliations:** 1 Department of Urology, People’s Hospital of Deyang City, 173#Northern Taishan Road, Deyang, 618000 China; 2 Department of Surgery, People’s Hospital of Deyang City, 173#Northern Taishan Road, Deyang, 618000 China; 3 Department of Laboratory, People’s Hospital of Deyang City, 173#Northern Taishan Road, Deyang, 618000 China

**Keywords:** FAGs, PRCC, Immune cell infiltration, TMB

## Abstract

**Background:**

This study aimed to identify the prognostic-related differentially expressed ferroptosis-associated genes (DEFAGs) in papillary renal cell carcinoma (PRCC).

**Methods:**

Data encompassing simple nucleotide variation, transcriptome profiles, and relevant clinical information of PRCC patients were sourced from The Cancer Genome Atlas (TCGA) database. The expression matrix of ferroptosis-associated genes (FAGs) was analyzed using the "limma" package in R to identify differentially expressed DEFAGs. Lasso regression analysis, along with univariate and multivariate Cox proportional hazards regressions, was employed to identify independent prognostic-related DEFAGs and formulate a nomogram. Additionally, we examined potential independent survival-related clinical risk factors and compared immune cell infiltration and tumor mutation burden (TMB) differences between high- and low-risk patient groups.

**Results:**

A cohort of 321 patients were analyzed, revealing twelve FAGs significantly influencing the overall survival (OS) of PRCC patients. Among them, two mRNAs (GCLC, HSBP1) emerged as independent prognostic-related DEFAGs. Smoking status, tumor stage, and risk score were identified as independent clinical risk factors for PRCC. Furthermore, notable disparities in immune cell infiltration and function were observed between high- and low-risk groups. GCLC and HSBP1 were associated with various immune cells and functions, TMB, and immune evasion.

**Conclusion:**

This finding revealed two independent prognostic-related DEFAGs in PRCC and established a robust prognostic model, offering potential therapeutic targets and promising insights for the management of this disease.

## Introduction

As one of the second most common renal cell carcinoma histological subtypes, papillary renal cell carcinoma (PRCC) accounts for 15 to 20% of renal cancers [1]. Although two tissue subtypes are recognized, their influence on prognosis remains controversial. However, it is well-documented that type 1 PRCC typically presents better survival rates compared to type 2 PRCC [2–5]. Currently, the treatment for PRCC, distinct from renal clear cell carcinoma (ccRCC), has been inadequate due to insufficient consideration and biomarkers, particularly for metastatic PRCC [6]. This, combined with the challenges of histological diagnosis, may contribute to a higher mortality rate.

Since it was first proposed by Stockwell et al. in 2012, ferroptosis, as a unique and emerging ferrous ion-dependent cell death form, has been gradually recognized for its potential physiological and pathological roles in tumor suppression and immune surveillance. Differing from apoptosis, cell necrosis, and autophagy, ferroptosis holds significant promise for immunotherapy [7–9]. This process of phospholipid peroxidation is intricately regulated by various cancer genes and signaling pathways, as well as being intertwined with cellular metabolism [10].

In the classic ferroptosis regulatory mechanism, two key cellular components, system-Xc, and GPX4, have been identified. Inhibiting these components triggers ferroptosis. GPX4, a selenoprotein, serves as the primary neutralizing enzyme for phospholipid hydroperoxides (PLOOHs), a form of reactive oxygen species (ROS) in cells. Furthermore, GPX4 facilitates the import of cystine into cells for cysteine production, as well as the generation of the potent reduced glutathione (GSH), thereby aiding in the reduction of PLOOHs [11, 12]. Compounds like erastin and RSL3 can directly or indirectly deactivate GPX4, hindering cystine entry into cells, depleting cellular antioxidant cysteine, and resulting in PLOOH accumulation, ultimately causing irreparable damage to the plasma membrane and cell death [13, 14].

Lipid peroxidation is intricately linked to the unsaturation degree of the lipid bilayer. The de-esterification of polyunsaturated fatty acyl moieties found on phospholipids (PUFA-PLs) is a potential initiator of lipid peroxidation [15]. Studies have highlighted acyl-CoA synthase long-chain family member 4 (ACSL4) as a critical player in ferroptosis, enhancing the integration of long-chain PUFAs into lipids and membrane structures [16, 17]. Inhibition of ACSL4 expression is considered to be a major mechanism of desensitization of cells to iron death [18, 19]. Meanwhile, enzymes like lipoxygenase (LOXs) or cytochrome P450 oxidoreductase (POR) can directly initiate ferroptosis through lipid peroxidation [20, 21]. In terms of cell metabolism, ferroptosis is also found to be related to metabolic events, such as autophagy and mitochondrial TCA cycle [22, 23]. Obviously, the homeostasis of iron in cells is also crucial, and iron regulatory proteins IRP1 and IRP2 can also regulate ferroptosis by changing the content of unstable iron in cells [24, 25]. GPX4-independent ferroptosis regulatory mechanisms have been proposed, including ferroptosis suppressor protein 1 (FSP1), GTP cyclohydrolase-1 (GCH1), and dihydroorotate dehydrogenase (DHODH) which protect tissues and cells from ferroptosis [26, 27].

In parallel with the progress of immunotherapy, immune checkpoint inhibitors (ICIs) have shown remarkable efficacy in various cancers, including renal and urothelial cancers, significantly enhancing the overall survival of select patients [28, 29]. Within the tumor microenvironment, immune cell infiltration can modulate and impact cancer progression, potentially serving as valuable biomarkers [30]. Furthermore, gene mutations in tumor cells may confer certain cells with the ability to evade immunity, escaping recognition or attack by the immune system [31]. The efficacy of immunotherapy may be linked to tumor mutation burden (TMB).

Ferroptosis, an iron-dependent form of regulated cell death characterized by the accumulation of lipid peroxides, has emerged as a significant factor in the progression of various cancers, including kidney renal papillary cell carcinoma (KIRP). Many studies have reported the biological significance of ferroptosis-related genes (FRGs) in diverse tumors, exploring their correlation with immune cell infiltration and tumor mutation burden [32, 33]. Our study focuses on FRGs and their implications in PRCC. The unique metabolic vulnerabilities of PRCC cells make them particularly susceptible to ferroptosis-inducing agents, which disrupt redox homeostasis and iron metabolism, leading to cancer cell death. This susceptibility is highlighted by our findings that specific FRGs, such as GCLC and HSBP1, play critical roles in modulating the ferroptotic pathway and are significantly associated with patient prognosis. The expression levels and the prognostic significance of these genes offer insights into the potential of targeting ferroptosis as a therapeutic strategy in PRCC, aiming to exploit the ferroptotic susceptibility of these tumors for improved clinical outcomes. Therefore, based on the data collected in the TCGA public database, we aim to evaluate the prognostic-related DEFAGs in PRCC.

## Materials and methods

### Data collection and preparation

Simple nucleotide variation, transcriptome, and associated clinical data of PRCC patients enrolled in the TCGA data portal before Sep 2021 were completely retrieved and extracted from the website (https://portal.gdc.cancer.gov/). Somatic mutation data were detected by Varscan, the “matfools” package of R was used to elucidate the mutation data. Samples with blank or unreported values or missing survival time would be excluded from clinical data. All sixty identified ferroptosis-associated genes (FAGs) were obtained from previous studies [52–55]. All statistical analyses were performed by R version 4.1.1 software. Since TCGA is an openly available dataset, no additional approval from an ethics committee was required.

### Identification and validation of prognostic ferroptosis-associated gene signatures

The expression matrix of FAGs was obtained by taking the intersection. The “limma” package of R was used to compare the differentially expressed FAGs (DEFAGs) of normal and tumor samples. Log2FC > 2 and FDR < 0.05 were considered statistically significant. Univariate Cox proportional hazards regressions were performed with the “survival” R package to calculate the prognostic-related FAGs. Subsequently, differentially expressed prognostic-related FAGs (prognostic-related DEFAGs) were identified through a combined analysis. Gene Ontology (GO) and Kyoto Encyclopedia of Genes and Genomes (KEGG) analyses were carried out using the "ClusterProfiler" and "org.Hs.eg.db" R packages, with a significance threshold set at P < 0.05 [54].

Lasso regression analysis and multivariate Cox proportional hazards regressions were conducted to minimize and screen the most significant independent risk factor DEFAGs related to the survival of patients with PRCC. Then, the nomogram was constructed based on these genes. According to the risk score, patients were also considered as high- or low-risk level. ROC and calibration curves of 3- and 5-year survival were used to verify the stability and accuracy of the model. Kaplan–Meier curves were employed to demonstrate the predictive capability of these model genes for overall survival (OS), with statistical significance set at P < 0.05.

### Multidimensional analysis of prognostic markers and clinical features

PCA was used to reduce the dimensionality of independent prognostic-related DEFAGs expression data in order to intuitively distinguish high- and low-risk groups. “c2.cp.kegg.v7.4.symbols.gmt” and “c5.go.v7.4.symbols.gmt” files were downloaded on the GSEA website (http://www.gsea-msigdb.org/). Then, active and enriched pathways or functions related to the independent prognostic-related DEFAGs set were revealed by GSEA. In addition, according to the clinical data (age, gender, race, tumor stage, smoking, BMI, and risk scores) obtained previously, we conducted analyses to explore potential independent clinical risk factors related to survival through univariate and multivariate Cox analyses.

### Tumor mutational burden (TMB) and mutation analysis for prognostic evaluation

TMB was quantified as the count of non-synonymous somatic variations per megabase (Mb) region, allowing for a comparison by assessing the disparity in mutant bases per million bases. To investigate differences in mutation burden, comparisons were made between the high- and low-risk groups of patients, and between the high- and low-expressed independent prognostic-related DEFAGs. These comparisons were visualized using box plots. Additionally, the mutation burden of prognostic-related FAGs was calculated. Furthermore, GSEA was conducted to unveil potential enriched functions associated with the high-risk group and the independent prognostic-related DEFAGs.

### Analysis of immune cell infiltration and immune escape mechanisms

Twenty-two types of immune cell fractions of each patient were estimated by the “CIBERSORT” R script. Single sample gene set enrichment analyses (ssGSEA) were used to get a corrected score of immune-related functions in each patient. According to the difference of high- and low-risk groups of patients and expression of selected independent prognostic-related DEFAGs, the difference and survival analyses of each immune cell and immune-related function were carried out, *P* < 0.05 was considered to be statistically significant. The patient’s immune escape potential was evaluated on the TIDE website (http://tide.dfci.harvard.edu). The differences of immune escape of patients between the high- and low-risk groups and the high- and low-expressed independent prognostic-related DEFAGs were also analyzed. Finally, the risk model was also compared with the prognostic models established based on the four TIDE scores (dysfunction, exclusion, MSI, and TIDE) and the 18 tumor inflammation signature (TIS) genes to verify its accuracy and stability [56].

## Results

### Prognostic significance of DEFAGs and their biological pathways

**Fig. 1:**
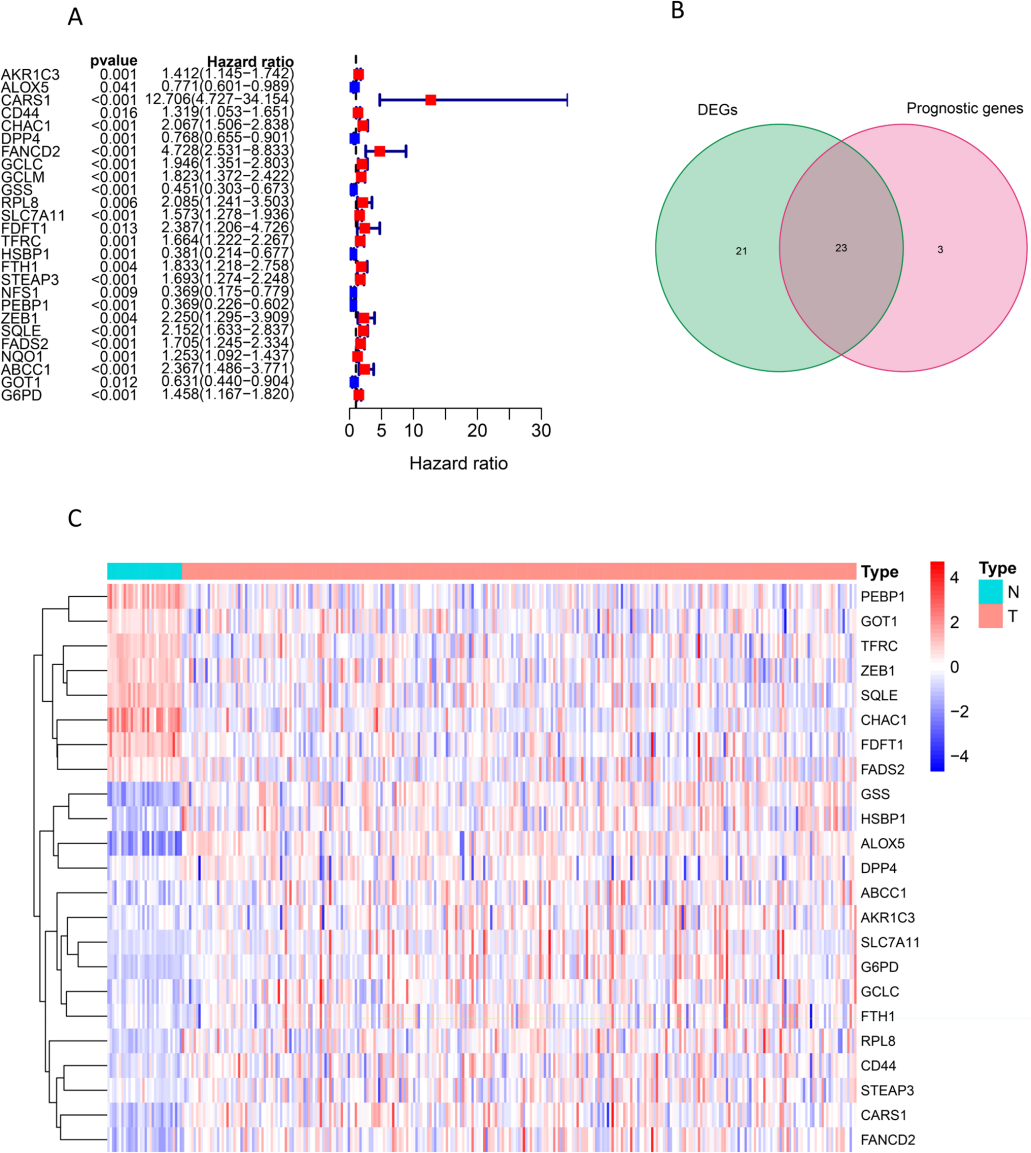
Identification of prognostic DEFAGs. Prognostic-related FAGs identified by the univariate Cox proportional hazards regressions (**A**); 23 prognostic-related DEFAGs obtained by an intersection (**B**); heatmap of the 23 prognostic-related DEFAGs (**C**)

**Fig. 2:**
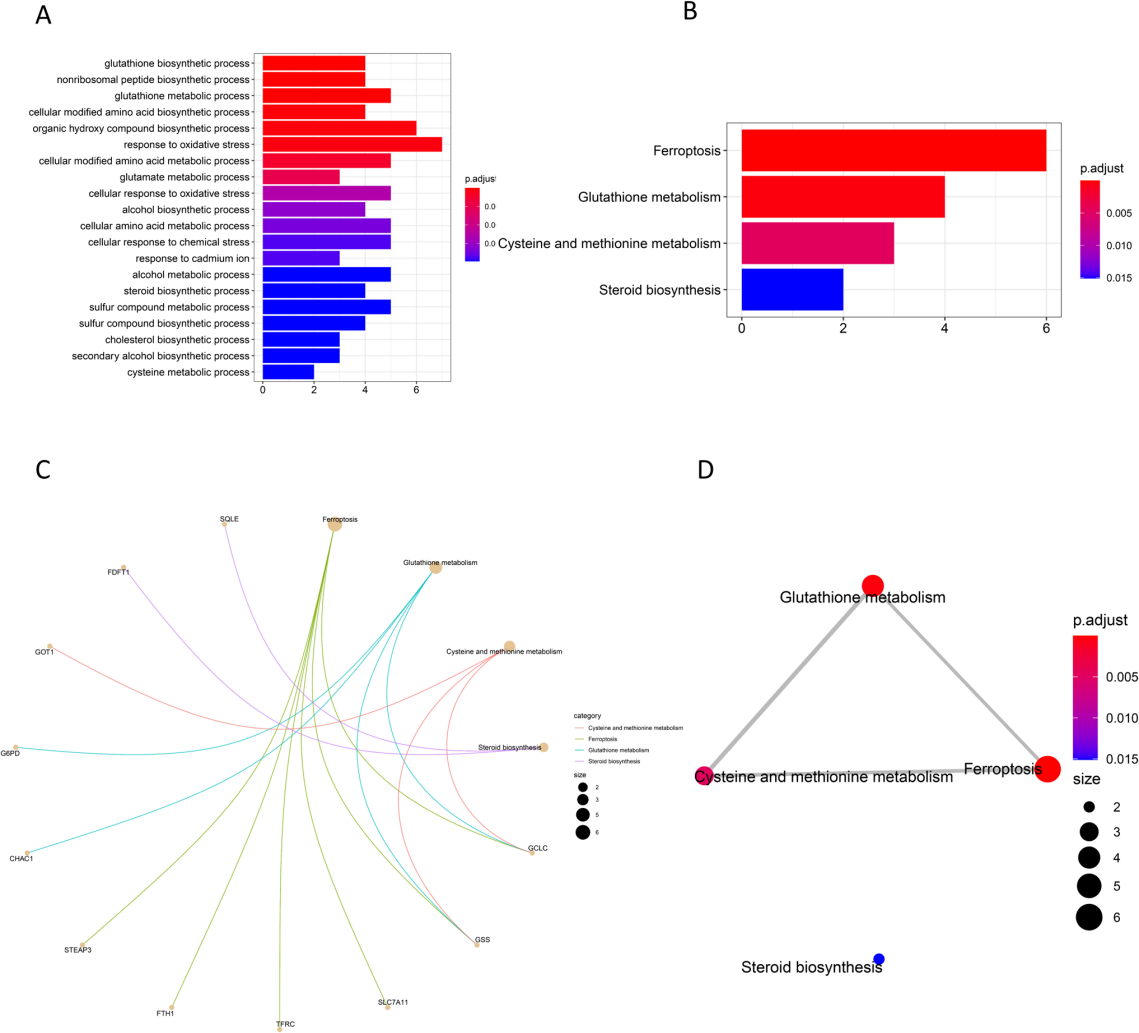
GO and KEGG analyses. GO (**A**) and KEGG (**B**) analysis, pathways–genes network (**C**) and pathways–pathways network (**D**) based on the 23 prognostic-related DEFAGs

The mRNAs expression matrix of the 321 patients (32 normal adjacent kidney tissue and 289 with PRCC) was collected in TCGA and clinicopathological characteristics of 191 complete patients were selected and presented in Table 1. A total of 60 mRNAs and their expression matrices were elucidated as FAGs. Among them, 44 significantly DEFAGs were evaluated using “edgeR” R packages, and 26 prognostic-related FAGs were identified after the univariate Cox proportional hazards regressions (P < 0.05) (Fig. 1A). Subsequently, 23 prognostic-related DEFAGs were obtained through an intersection analysis (Fig. 1B), with their expression patterns visualized in heatmaps (Fig. 1C). GO analysis showed that these 23 prognostic-related DEFAGs were primarily associated with processes, such as glutathione biosynthesis, glutathione metabolism, non-ribosomal peptide biosynthesis, cellular modified amino acid biosynthesis, and organic hydroxy compound biosynthesis (Fig. 2A). The top four KEGG pathways enriched by these genes included ferroptosis, glutathione metabolism, cysteine and methionine metabolism, and cysteine and methionine metabolism (Fig. 2B). Furthermore, eleven genes among the 23 prognostic-related DEFAGs were related with the four pathways (Fig. 2C), and a connection between these pathways was existing (Fig. 2D).

**Table 1:** Clinicopathological characteristics of PRCC patients

	Total (*n* = 191)	%	Alive (*n* = 157)	%	Dead (*n* = 34)	%
Age at diagnose						
< 60 (years)	73	38.2	60	38.2	13	38.2
60–79 (years)	105	55.0	84	53.5	21	61.8
≥ 80 (years)	13	6.8	13	8.3	0	0
Gender						
Male	140	73.3	118	75.2	22	64.7
Female	51	26.7	39	24.8	12	35.3
Race						
Asian	3	1.6	2	1.3	1	2.9
White	133	69.6	107	68.1	26	76.5
Others	55	28.8	48	30.6	7	20.6
Tumor clinical stage						
Stage I	128	67.1	117	74.5	11	32.4
Stage II	14	7.3	12	7.6	2	5.9
Stage III	35	18.3	22	14.1	13	38.2
Stage IV	14	7.3	6	3.8	8	23.5
Smoking						
Yes	58	30.4	53	33.8	5	14.7
No	133	69.6	104	66.2	29	85.3
BMI						
< 25	46	24.1	37	23.6	9	26.5
25–40	135	70.7	112	71.3	23	67.6
≥ 40	10	5.2	8	5.1	2	5.9
Mean (SD) (days)	1015.17 ± 893.22		1026.2 ± 922.3		964.24 ± 754.47	
Median[MIN, MAX] (days)	762.50		757.00		697.00

### Identification of independent prognostic DEFAGs and development of a predictive nomogram for PRCC

**Fig. 3:**
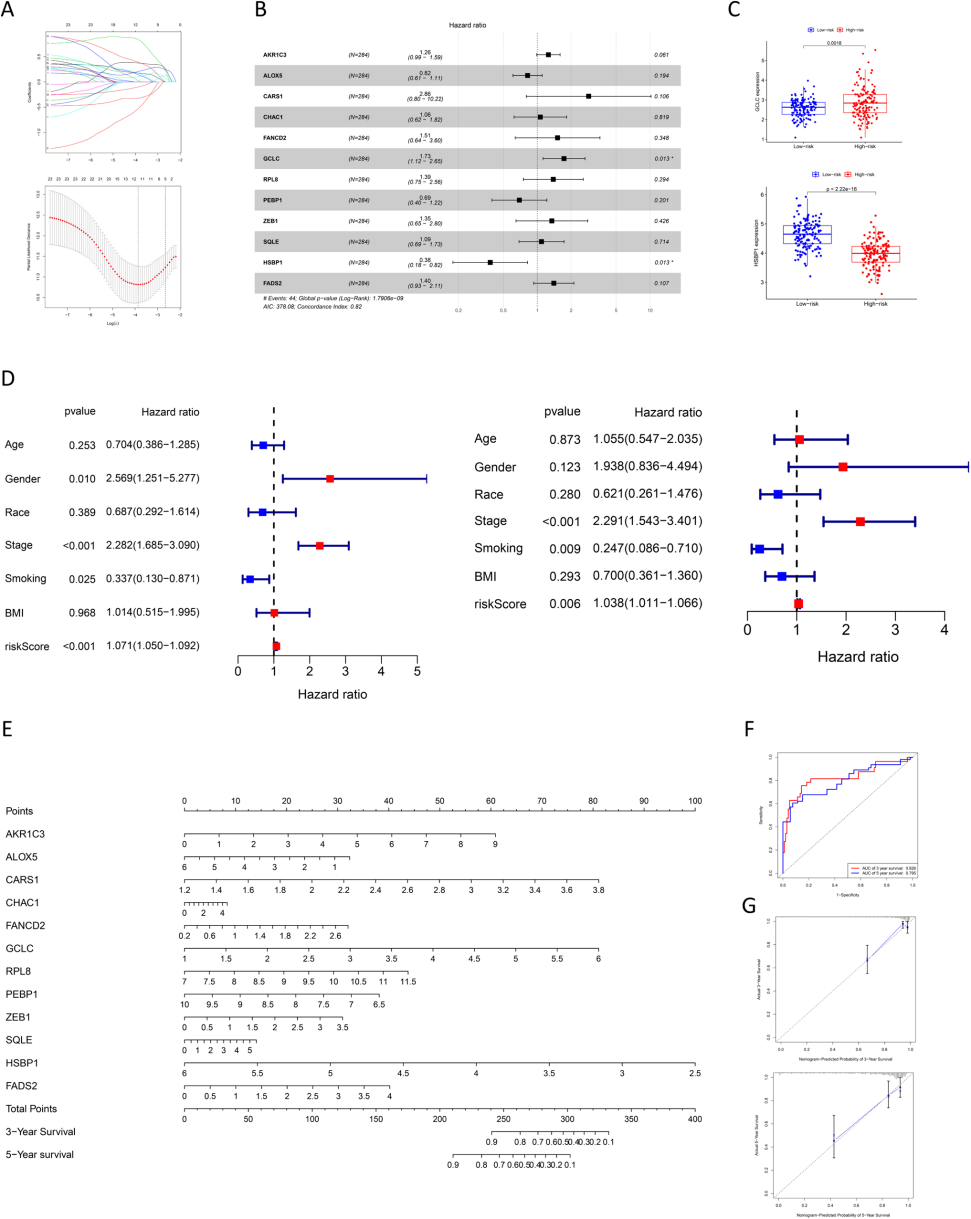
DEFAGs and nomogram. Lasso regression analysis (**A**) and subsequent multivariate Cox proportional hazards regressions (**B**) of the prognostic-related DEFAGs; expression of GCLC and HSBP1 in high- and low-risk group (**C**); univariate and multivariate prognostic analyses based on the patients’ clinical characteristics (**D**); nomogram based on the genes selected by lasso analysis (**E**); 3- and 5-year receiver operating characteristic curve (**F**) and calibration curve (**G**) of the regress model

**Fig. 4:**
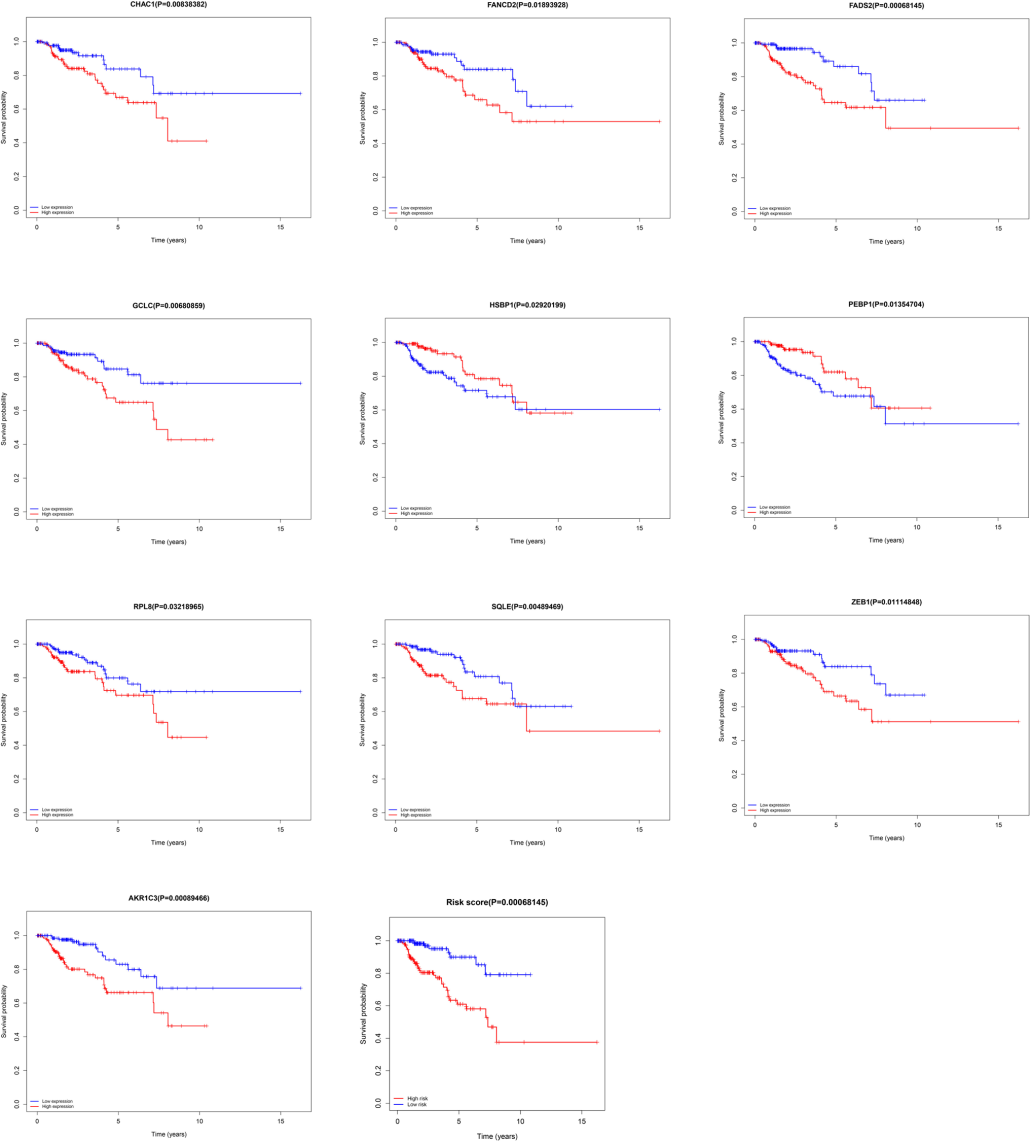
Kaplan–Meier analyses based on the risk score and the genes selected by lasso analysis (*P* < 0.05)

Lasso regression analysis and multivariate Cox proportional hazards regressions identified 2 independent prognostic-related DEFAGs (GCLC and HSBP1) in PRCC patients (Fig. 3A, B). Patients were stratified into high- and low-risk groups based on their risk scores, with GCLC and HSBP1 showing distinct expression patterns in these groups (Fig. 3C). Univariate and multivariate prognostic analyses were conducted on patients' clinical characteristics, revealing that smoking, tumor stage, and risk score were independent risk factors (Fig. 3D). A nomogram was developed based on the 12 genes (AKR1C3, ALOX5, CARS1, FANCD2, GCLC, RPL8, PEBP1, ZEB1, SQLE, HSBP1, FADS2) identified after lasso analysis (Fig. 3E). The AUC (area under the receiver operating characteristic curve) for 3- and 5-year survival was 0.826 and 0.795, respectively, confirming the model's reliability and accuracy (Fig. 3F). Calibration curves also demonstrated strong consistency (Fig. 3G). Kaplan–Meier analyses revealed that all 12 genes identified post-lasso analysis were significantly associated with the overall survival (OS) of PRCC patients (P < 0.05) (Fig. 4).

### Divergence in genetic profiles and pathway enrichment between PRCC risk groups

**Fig. 5:**
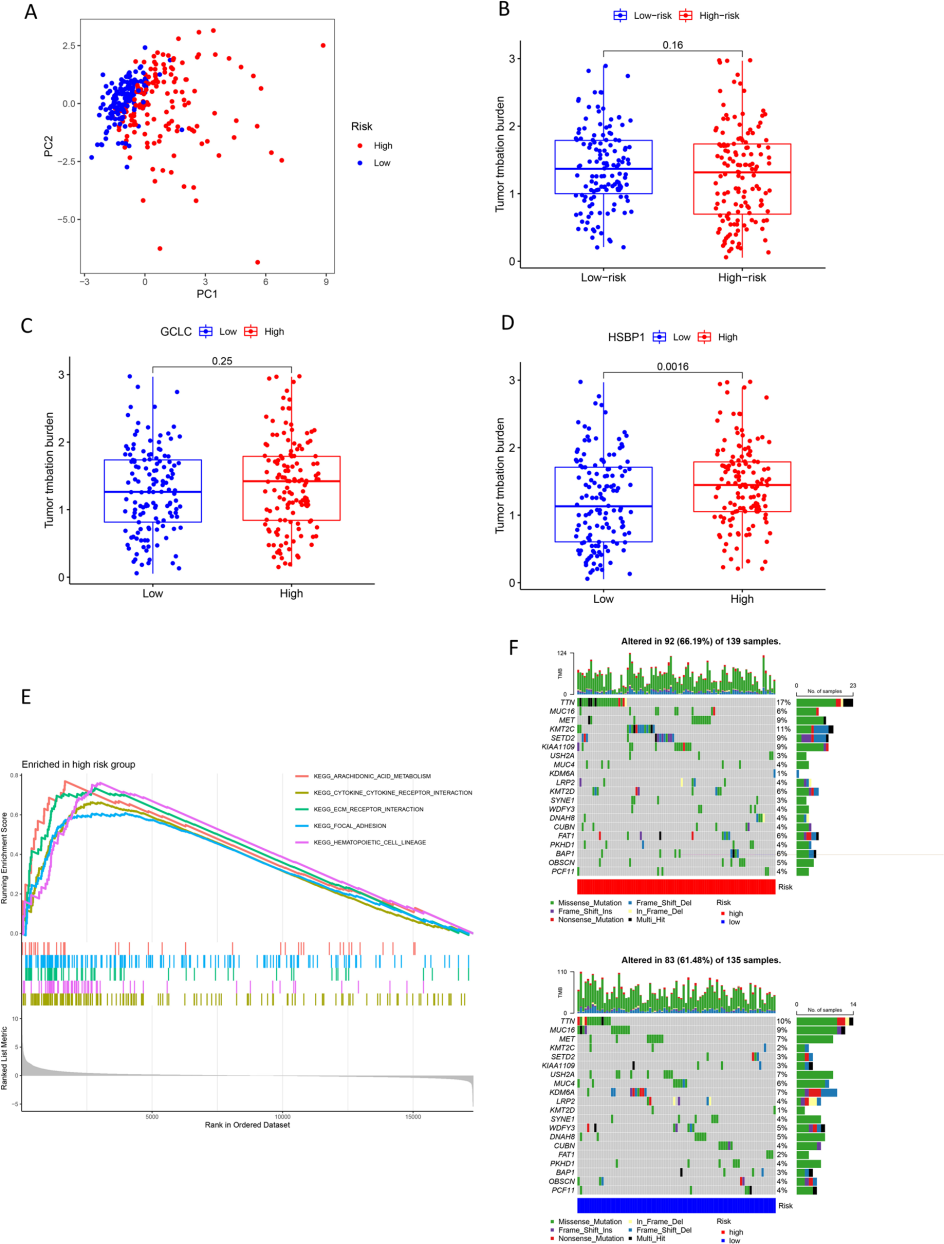
PCA, gene mutation, and enrichment. PCA of independent prognostic-related DEFAGs (**A**); difference of TMB between high- and low-risk group (**B**); difference of TMB of HSBP1 (**C**) and GCLC (**D**); GSEA of high-risk group (**E**); gene mutation in the high- and low-risk group (**F**)

PCA showed significant differences between high- and low-risk groups (Fig. 5A). While there was no significant difference in tumor mutation burden (TMB) between the groups (Fig. 5B), the expression levels of HSBP1 were higher in samples with elevated TMB, whereas GCLC expression did not vary significantly with changes in TMB (Fig. 5C, D). Gene set enrichment analysis (GSEA) of high-risk patients highlighted enrichment in pathways, such as arachidonic acid metabolism, cytokine receptor interaction, ECM receptor interaction, focal adhesion, and hematopoietic cell lineage (Fig. 5E). These enriched pathways suggest a potential mechanistic explanation for the more aggressive tumor behavior observed in the high-risk group. Mutation analysis revealed distinct mutation profiles in the high- and low-risk groups, with TTN and KMT2C being the most frequently mutated genes in the high-risk group, and TTN and MUC16 in the low-risk group (Fig. 5F). This analysis provides a deeper understanding of the genetic variations that may contribute to the differential risk and potentially influence the prognosis and therapeutic responses in PRCC patients.

### Impact of immune cell dynamics on survival and immune escape in PRCC risk groups

**Fig. 6:**
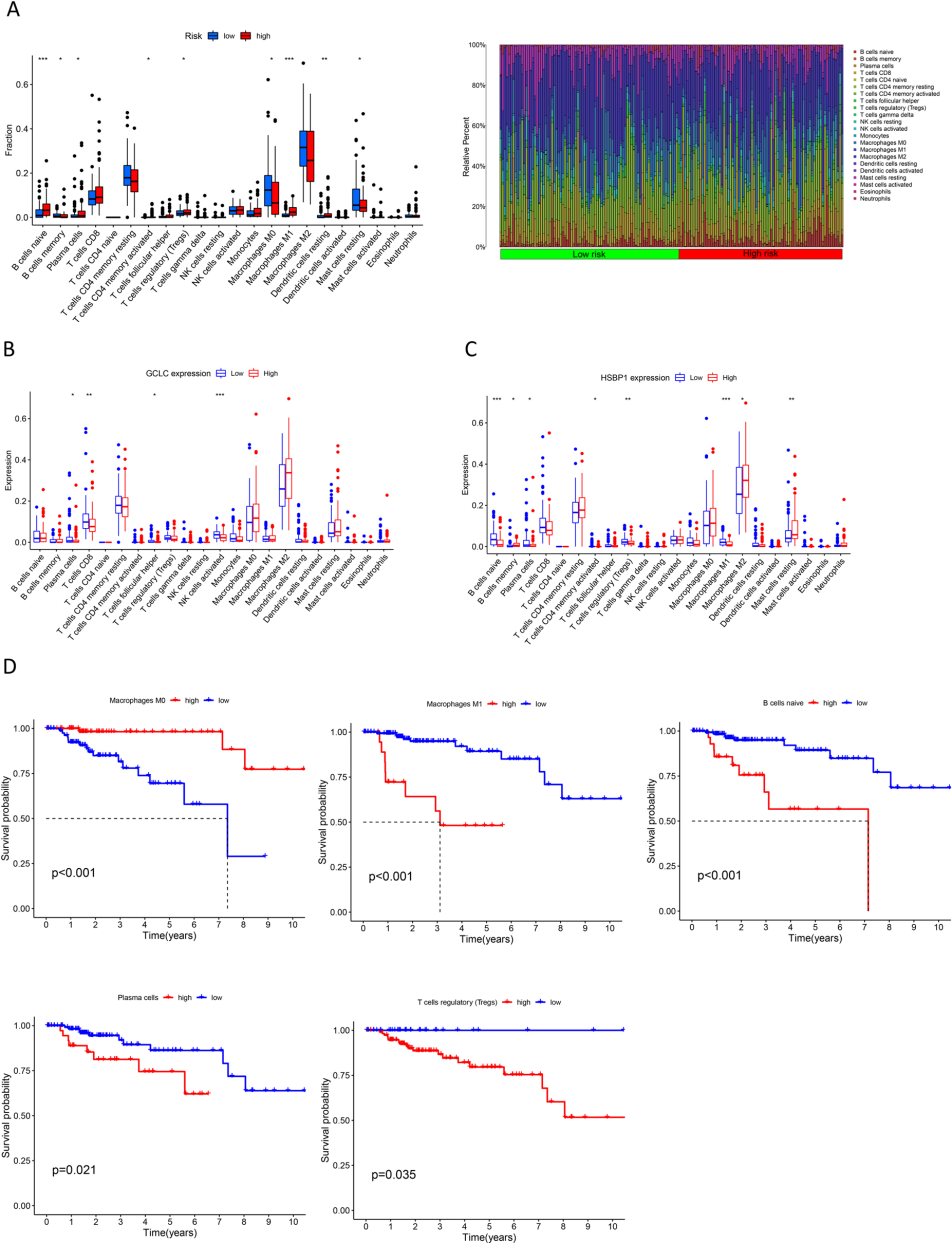
Immune cell analysis and immune escape. Difference analyses of the 22 immune cells between high- and low-risk group (**A**); Difference analysis of the 22 immune cells related to the expression of GCLC (**B**) and HSBP1 (**C**); survival analyses the 22 immune cells (**D**)

**Fig. 7:**
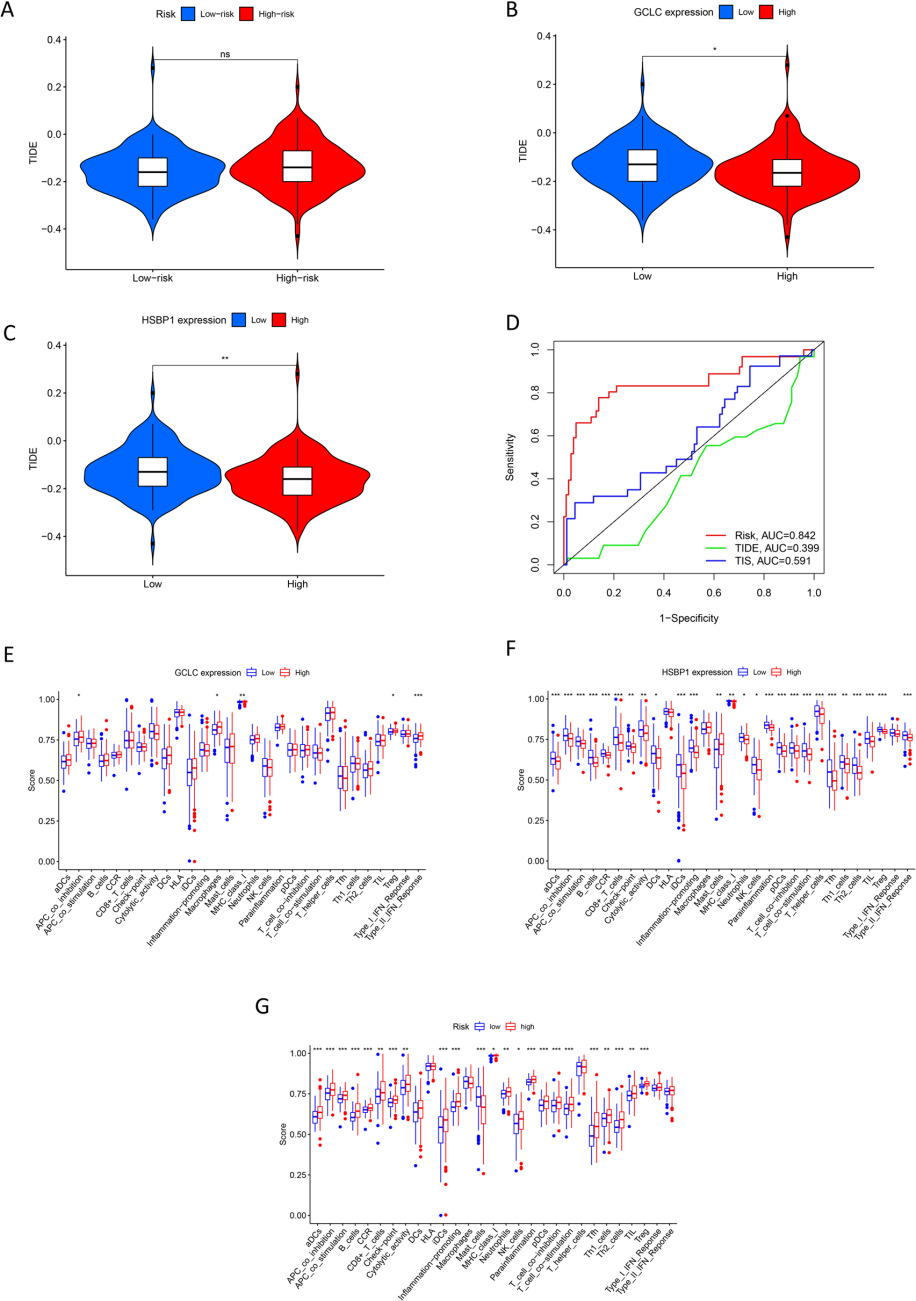
TIDE score, model verification, and immune function analysis. Differences of TIDE score between the high- and low-risk groups (**A**); Differences of TIDE score based on the expression of GCLC (**B**) and HSBP1 (**C**); ROC of the risk, TIDE, and TIS model (**D**); difference analyses of immune-related functions between high- and low-risk group (**E**); difference analyses of immune-related functions related to the expression of GCLC (**F**) and HSBP1 (**G**)

Nine out of 22 immune cell types, including B cells naïve, B cells memory, plasma cells, T cells CD4 memory-activated, T cells regulatory (Tregs), macrophages M0, macrophages M1, dendritic cells resting, and mast cells resting, exhibited significant differences between the high- and low-risk groups (Fig. 6A). Plasma cells, T cells CD8, T cells follicular helper, and activated NK cells showed significant associations with the expression of GCLC (Fig. 6B). Furthermore, B cell-naïve, B cell memory, plasma cells, T cells CD4 memory-activated, Tregs, macrophages M1, macrophages M2, and mast cells resting were linked to the expression of HSBP1 (Fig. 6C). A decrease in macrophages M0 was indicative of a poorer prognosis for patients (P < 0.01), whereas macrophages M1, plasma cells, Tregs, and B cell-naïve showed the opposite trend (Fig. 6D). The high-risk group displayed a modest benefit in terms of tumor immune dysfunction and exclusion (TIDE), an analytical tool used to predict immune escape and the potential efficacy of immune checkpoint inhibitors based on tumor genomics [34]. This metric is crucial in evaluating the likelihood of a tumor's response to immunotherapy (Fig. 7A). Notably, it seemed that elevated expression of GCLC and HSBP1 was associated with lower TIDE scores (Fig. 7B, C).

### Efficacy of the risk model in predicting immune function impact and prognosis in PRCC

**Fig. 8:**
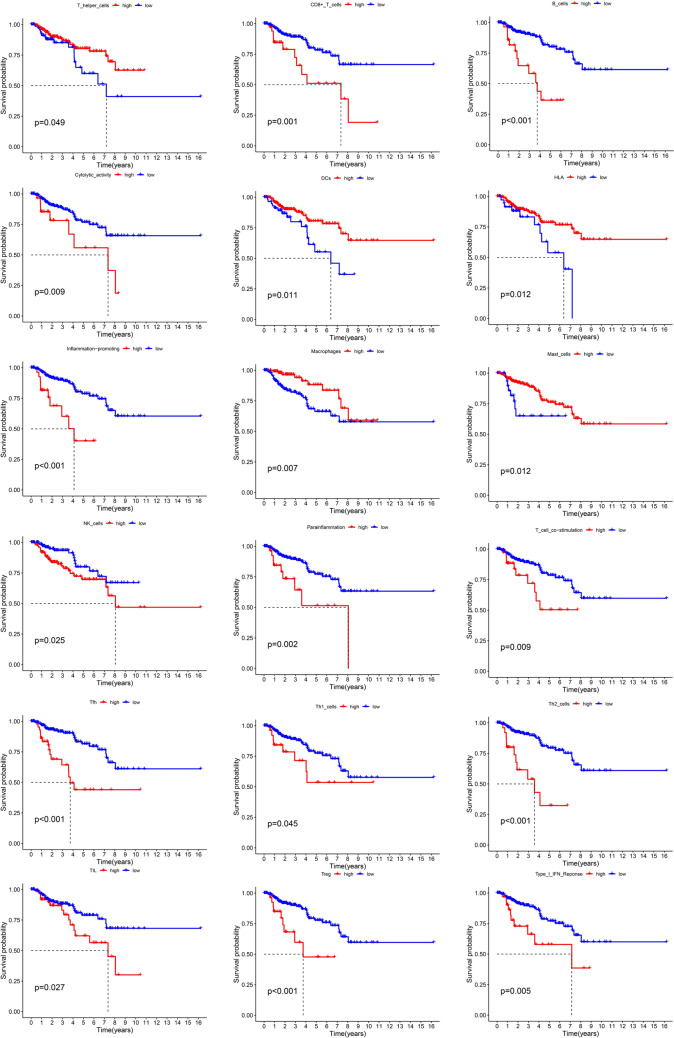
Survival analysis of immune-related functions

The risk model demonstrated superior performance in terms of AUC compared to the TIDE or TIS models, highlighting its accuracy and reliability (Fig. 7D). High expression of GCLC showed positive correlations with APC co-inhibition, macrophages, MHC class I, Tregs, and type II IFN response (Fig. 7E). Similarly, the expression of HSBP1 was linked to variations in multiple immune functions (Fig. 7F). Significant differences were observed between the high- and low-risk groups in various immune functions, excluding DCs, HLA, macrophages, T helper cells, and type I/II IFN response (Fig. 7G), all of which were also associated with patient prognosis (Fig. 8).

## Discussion

Since the commencement of the ferroptosis concept, a plethora of studies have underscored the significant role of ferroptosis in cancers, degenerative diseases, and ischemic organ damage. This iron-dependent, phospholipid peroxidation-driven form of programmed cell death is intricately linked to various cellular metabolic processes and signaling pathways, encompassing lipid, amino acid, and sugar metabolism, iron regulation, redox balance, and mitochondrial activity [10].

Building upon the known mechanisms of ferroptosis, a spectrum of enzymes regulating ferroptosis are emerging as promising therapeutic targets. Robert et al. suggested that disrupting the SLC7A11 subunit of system-Xc could reduce cellular cystine levels, triggering ferroptosis [35, 36]. Previous studies have indicated elevated SLC7A11 expression in renal chromophobe cell carcinoma, potentially subject to competitive inhibition by upstream lncRNA [37]. It suggests that renal chromophobe cancer cells heavily rely on Xc-system-generated cystine to combat reactive oxygen species (ROS) and evade ferroptosis, making SLC7A11 inhibition a valuable therapeutic avenue [38]. FSP1 is also recognized as a significant therapeutic target. Studies have shown that inhibiting FSP1 can induce ferroptosis in cancer cells with GPX4 deficiency or overexpression. Given its prevalent expression across most cancers, FSP1 holds substantial clinical utility [39]. The immune system also plays a role in targeting key nodes within the ferroptosis process. Research indicates that immune cells can modulate ferroptosis by suppressing GPX4 activity [40]. Recent investigations into the tumor microenvironment and the mechanisms of cancer cell death have highlighted the potential synergy between ferroptosis and immunotherapy, particularly in enhancing cancer treatment efficacy. For instance, research has shown that T cell-derived interferon-gamma (IFNγ), when combined with arachidonic acid (AA), prompts ferroptosis in tumor cells by upregulating ACSL4 and altering lipid compositions, thereby sensitizing them to iron-dependent cell death [41]. This pathway not only serves as a natural mechanism for CD8 + T cell-mediated tumor eradication but also underlines the potential therapeutic value of targeting ferroptosis in conjunction with immunotherapy. Furthermore, in hepatocellular carcinoma (HCC), single-cell RNA sequencing has unearthed the pivotal role of APOC1 in modulating the tumor-associated macrophage (TAM) phenotypes from pro-tumor M2 to pro-inflammatory M1 through the ferroptosis pathway. Inhibition of APOC1 demonstrated a significant reduction in tumor progression and an altered immune cell profile favorable for enhancing the efficacy of anti-PD-1 immunotherapy [42]. Additionally, the negative correlation between APOC1 expression and PD-1/PD-L1 levels in human HCC samples suggests that manipulating ferroptosis pathways could further refine the responsiveness to immunotherapeutic agents. These findings collectively underscore the profound impact of ferroptosis inducers in reshaping the immune landscape, offering a robust strategy for augmenting the response to immunotherapy across various cancer types.[35].

In current study, the biological value of the two overexpressed independent prognostic-related DEFAGs (GCLC, HSBP1) was systematically analyzed by lasso regression analysis, univariate and multivariate Cox proportional hazards regressions. The catalytic subunit of glutamate–cysteine ligase (GCLC) could inhibit ferroptosis by participating in the first step of GSH synthesis. Recent research has shown that even in cysteine-depleted conditions, GCLC can be regulated by NRF2, leading to increased γ-glutamyl peptides, ultimately reducing intracellular glutamate levels and resisting ferroptosis [43]. Various studies have found out that high expressed GCLC was associated with the poor prognosis and drug resistance of a variety of cancers [44–46]. Nguyen A et al. also reported that GCLC was overexpressed in liver metastases [47]. Our study also showed that patients in the high-risk group exhibited higher GCLC expression and worse overall survival. Additionally, HSBP1 was reported to be overexpressed in ovarian cancer and may be regulated by corresponding lncRNAs [48]. It has been reported that HSBP1 is highly expressed and may be regulated by Lin28A. Lin28A can enhance the mRNA stability and protein expression of HSBP1, which is significantly correlated with poor survival outcomes in ovarian cancer patients [48]. This regulation occurs via the enrichment of HSBP1 mRNA in the RNA-induced silencing complex (RISC) loaded with Lin28A, indicating a critical post-transcriptional regulatory role by Lin28A. Moreover, RAN and HSBP1, when knocked down in ovarian cancer cells with high Lin28A expression, resulted in reduced malignancy characteristics, such as cell survival, invasion, and tumor growth in vivo, alongside increased apoptosis rates. However, these findings may appear discrepant with our results, which did not observe a similar regulatory mechanism or expression pattern in our cancer type. The potential discrepancy can be attributed to several factors. First, the tissue-specific expression and regulatory mechanisms of HSBP1 may differ significantly between ovarian cancer and PRCC. Ovarian cancer cells might possess unique regulatory pathways influenced by specific microenvironmental factors not present in other types of cancer. Second, the role of Lin28A in regulating HSBP1 could be specific to ovarian cancer due to its involvement in maintaining stemness characteristics, which are more pronounced in ovarian cancer stem cells. Third, the molecular background of the patients, including differences in genetic, epigenetic, and transcriptional landscapes, can lead to diverse expression and regulation of the same genes across different cancers.

Multivariate regression analysis of the clinical characteristics of patients showed that stage, smoking, and risk score were independent risk factors that affected the prognosis of PRCC patients, which proved the reliability and accuracy of our model. Interestingly, unlike renal clear cell carcinoma, BMI was no longer an independent risk factor for PRCC. Significant differences in multiple immune cells and their functions were observed between the high- and low-risk groups, significantly impacting PRCC patients' prognosis. While our study found no significant disparity in tumor mutational burden (TMB) and TIDE between the high- and low-risk groups, which may suggest challenges in predicting the efficacy of immune checkpoint inhibitors (ICIs) in PRCC, it is important to acknowledge that ICIs have demonstrated success in several clinical cases of PRCC. This underscores the complex interplay of immune functions and highlights the need for further investigation to fully understand the predictors of response to ICIs in this specific cancer subtype [49–51]. Notably, GCLC and HSBP1 appeared to play active roles in regulating the tumor microenvironment and immune responses in our study. High expression of HSBP1 might also enhance the efficacy of ICIs by preventing tumor immune escape and increasing immune mutation burden.

The identification of the two independent prognostic-related DEFAGs in PRCC is particularly significant, as it contrasts with their roles observed in other types of cancer, indicating unique mechanisms at play in PRCC that may differ from those in other malignancies. However, our study has several limitations. First, the risk model has not been validated in other databases or with local patients. Secondly, there is a lack of in vivo and in vitro experiments to verify the function and specific mechanisms of the independent prognostic-related DEFAGs. Nonetheless, their biological significance in PRCC patients warrants acknowledgment.

## Conclusion

Our study has identified two independent prognostic-related DEFAGs (GCLC, HSBP1) in PRCC and has effectively developed a robust prognostic model. Additionally, we evaluated variances in immune cell infiltration, tumor mutation burden, and TIDE scores between the high- and low-risk groups, along with the expression levels of GCLC and HSBP1. These findings are poised to provide novel perspectives on the prognosis and treatment of PRCC.

## Data Availability

All datasets analyzed in this study are available online, which can be found at the websites below: https://portal.gdc.cancer.gov/. Raw data of our study are shared on the Nutstore: https://www.jianguoyun.com/p/DQpFWfYQ3qecCxjcrOwEIAA
